# Supplementary material for: P2K1 Receptor, Heterotrimeric Gα Protein and CNGC2/4 Are Involved in Extracellular ATP-Promoted Ion Influx in the Pollen of Arabidopsis thaliana
Source: Plants (Basel). 2021 Aug 23;10(8):1743. doi: 10.3390/plants10081743 (PMC8400636; doi:10.3390/plants10081743)
Supplement: Supplementary file 1 [file plants-10-01743-s001.zip › plants-1345720-supplementary.pdf]

plants

MDPI

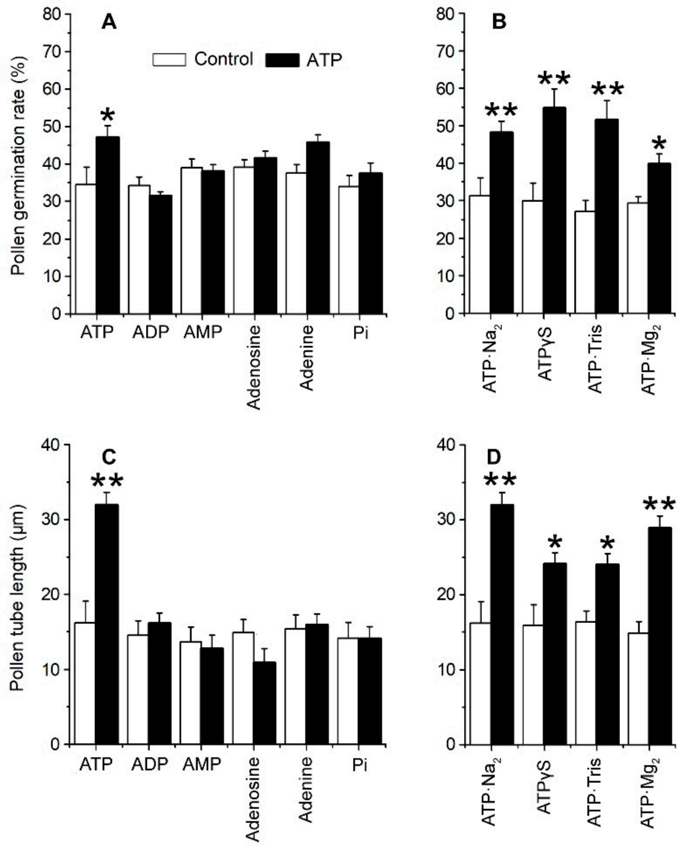

Figure S1. The effects of ATP, ATP agonist, or ATP hydrolytes on PG and PTG in *Arabidopsis thaliana.* A-D show the effects of 0.1 mM ATP (sodium-, magnesium- or Tris- salt), weakly hydrolysable ATP agonist (ATPγS), and ATP hydrolytes (ADP, AMP, adenosine or adenine) on PG (**A**, **B**) and PTG (**C**, **D**). KCl concentration in the medium was 0.1 mM. In each experiment, at least 300 pollen grains or 150 pollen tubes were counted or measured. Data from 3 replicates were combined to obtain mean±SD. Student's *t*-test *p* values: **p*<0.05,***p*<0.01.

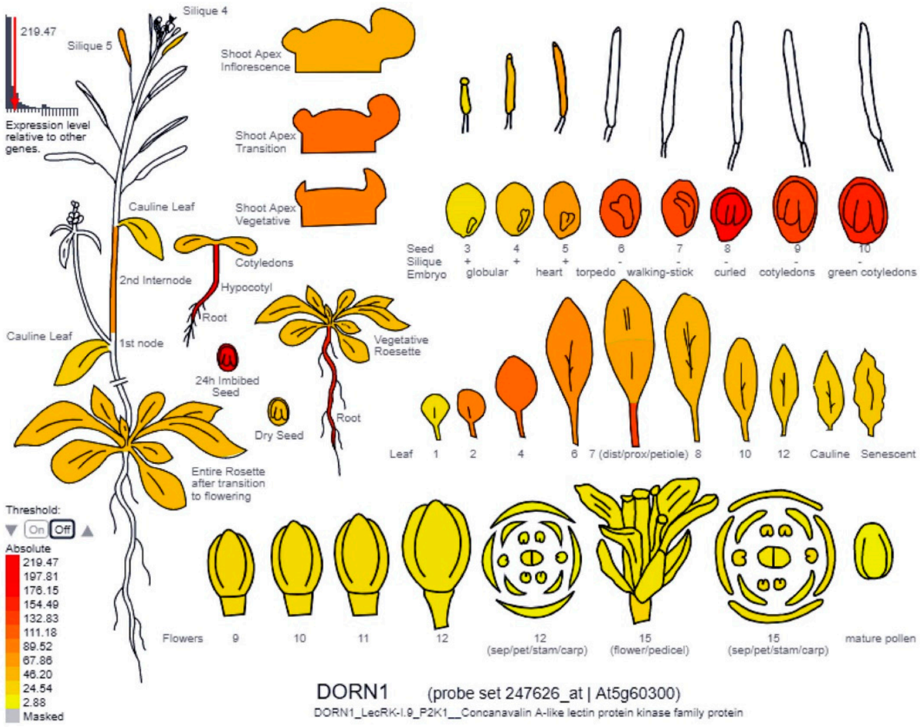

Figure S2. The expression pattern of DORN 1 in *Arabidopsis thaliana*. Data are from the information on website of the Arabidopsis Information Resource. **(**http://bar.utoronto.ca/~jwaese/Arabidopsis/Arabidopsis_ eFPBrowser2.html? primaryGene=DORN1**)**
